# Importance of IGF-I levels in IVF: potential relevance for growth hormone (GH) supplementation

**DOI:** 10.1007/s10815-021-02379-8

**Published:** 2022-01-23

**Authors:** Norbert Gleicher, Sarah K. Darmon, Emanuela Molinari, Pasquale Patrizio, David. H. Barad

**Affiliations:** 1 The Center for Human Reproduction, 21 East 69th Street, New York, NY 10021 USA; 2 The Foundation for Reproductive Medicine, New York, NY USA; 3 Stem Cell Biology and Molecular Embryology Laboratory, The Rockefeller University, New York, NY USA; 4 Department of Obstetrics and Gynecology, Medical University of Vienna, Vienna, Austria; 5 Department of Obstetrics, Gynecology and Reproductive Sciences, Yale University, New Haven, CT USA

**Keywords:** Insulin-like growth factor (IGF-1), IVF, In vitro fertilization, Cycle cancellations, Infertility

## Abstract

**Purpose:**

Growth hormone (GH) supplementation in association with in vitro fertilization (IVF) is worldwide again increasing, even though study outcomes have been discrepant. Since GH acts via insulin-like growth factor-1 (IGF-1), its utilization in IVF would only seem to make sense with low IGF-1. We, therefore, determined whether IGF-I levels affect IVF outcomes.

**Methods:**

Retrospectively, 302 consecutive first fresh, non-donor IVF cycles were studied, excluding patients on GH supplementation. Patients were divided into 3 subgroups: IGF-1 in lower 25th percentile (group A, < 132 ng/mL, *n* = 64); 25th–75th percentile (B, 133–202 ng/mL, *n* = 164), and upper 25th percentile (C, > 202 ng/mL, *n* = 74). IGF-1 was tested immunochemiluminometric with normal range at 78–270 ng/mL. Because of the study patients’ adverse selection and low pregnancy chances, the main outcome measure for the study was cycle cancellation. Secondary outcomes were oocyte numbers, embryos transferred, pregnancies, and live births.

**Results:**

Group A was significantly older than B and C (*P* = 0.019). IGF-1 decreased with increasing age per year by 2.2 ± 0.65 ng/mL (*P* = 0.0007). FSH was best in group B and worst in A (trend, *P* = 0.085); AMH was best in B and worst in A (N.S.). Cycle cancellations were lowest in C (11.6%) and highest in A (25.0%; *P* = 0.042). This significance further improved with age adjustment (*P* = 0.021). Oocytes, embryo numbers, pregnancies, and live birth rates did not differ, though oocyte numbers trended highest in B.

**Conclusions:**

Here presented results support the hypothesis that IGF-1 levels affect IVF outcomes. GH treatments, therefore, may be effective only with low IGF-1.

## Introduction

As add-on to ovulation induction for intrauterine inseminations [1] and in vitro fertilization (IVF) stimulation protocols [2], growth hormone (GH) supplementation was actively utilized for a little over a decade starting in the late 1980s. After a relative hiatus of approximately two decades, GH supplementation has in the last 15 years again become more fashionable [3, 4], even though effectiveness of GH supplementation in improving IVF outcomes has remained controversial [5, 6].

GH is a peptide hormone secreted by anteriorly positioned cells in the pituitary gland (somatotrophs) and plays multiple important roles in the body which go far beyond just support of linear growth, as its name would suggest. Released in pulsatile fashion by GH-releasing hormone with peaks during sleep, it is inhibited by somatostatin, produced in the hypothalamus. Its levels are the highest during puberty and are affected by environmental factors, like sleep patterns, diet, exercise habits, and exposure to stress. The hormone’s principal organ target is the liver, where it induces synthesis of insulin-like growth factor (IGF-1) [7]. GH’s principal (though not only) activity, therefore, is mediated by IGF-1. How GH and IGF-1 affect reproductive tissues has recently been reviewed [8].

Though thus a good number of studies have investigated GH-supplementation in conjunction with IVF, peripheral IGF-1 values in infertile women have been only minimally explored and, indeed, with contradictory findings by the same institution [9, 10].^.^ Some studies have reported on IGF-1 in follicular fluids and observed correlations to IVF outcomes [11–13].

The GH/IGF-1 signaling pathway (at times also called the somatotropic axis) relates strongly to aging [12, 13]. In centenarians, functional mutations in the IGF-1-receptor (*IGF-1R*), resulting in diminished IGF-1 signaling, are enriched [14, 15]. In women, low IGF-1 was demonstrated to offer a general survival advantage [16]. As of this point, effects of IGF-1 on ovarian aging are not well defined. Animal data, however, have convincingly demonstrated that GH can stimulate IGF-1 secretion not only from the liver but also from peripheral organs, including ovaries. To complicate matters further, such local IGF-1 secretion can also be stimulated by steroid hormones and/or gonadotropins. Moreover, GH can also be produced locally in the ovary, in which case the hormone functions in a paracrine, no-pulsatory, and non-circadian mode without involvement of the GH receptor (*GHR*) [17].

A mouse model, knockout of GHR, however, interestingly did *not* prevent fertility but reduced litter size [18], thereby delaying exhaustion of the follicle pool [13]. Diminished GH activity in the ovary may, thus, help in maintaining the resting follicle pool (i.e., reduce recruitment), as it naturally declines with advancing female age (i.e., declining functional ovarian reserve). This is also supported by histological examinations, demonstrating a decline in the growing follicle pool. That IGF-1 is, likely, involved in the signaling cascades for these observations is demonstrated by the fact that IGF-1 administration reverses them [19]. Moreover, knockout of the IGF-1 gene in the mouse does results in infertility (and dwarfism), a phenotype that cannot be rescued with gonadotropin stimulation and on histology demonstrates a complete arrest in the development of the growing follicle pool [17]. IGF-1, thus, appears essential for follicle maturation.

Hsu and Hammond in 1987 were the first to demonstrate that GH increased ovarian IGF-1 production in granulosa cells, thereby enhancing FSH action [20]. We today know that GH and androgens share in this function at small growing follicle stages [21]. With increasing clinical utilization of GH supplementation in IVF, a better understanding of IGF-1 effects on ovaries appears, however, urgently needed. For example, GH supplementation would appear senseless in presence of normal or even high IGF-1 levels, as any hormone supplementation only appears indicated if concentrations in the to be treated microenvironment are insufficient. It indeed would not surprise if above noted persisting controversy whether GH supplementation improves IVF outcomes may be due to unselected indiscriminate utilization of such supplementation in infertile women. Assuming normal endocrine physiology, GH supplementation should only be effective in women with abnormally low IGF-1 levels.

To elucidate the potential importance of peripheral IGF-1 levels for IVF outcomes, this study, therefore, investigated the importance of untreated initial peripheral IGF-1 levels on IVF cycle outcomes. Results support the hypothesis that peripheral IGF-1 levels relate to IVF cycle outcomes and, therefore, suggest that GH supplementation should only be applied selectively.

## Materials and methods

### Study population

We report on 978 consecutive patients undergoing 815 IVF cycles at our center between 2018 and 2020 who as part of a diagnostic work-up had peripheral IGF-1 level determinations at time of initial consultation. Bloods were routinely obtained approximately 6–8 weeks before IVF cycle start. Patients on GH supplementation and/or in repeat IVF cycles at our center were excluded from this study. Ultimately, 302 fresh first non-donor cycles qualified for the study. Based on IGF-1 levels, these women were then divided into 3 subgroups representing the lower 25th percentile (group A, < 132 ng/mL, *n* = 64), the 25th–75th percentile (group B, 132–202 ng/mL, *n* = 164), and the upper 25th percentile (group C, > 202 ng/mL, *n* = 74), with A considered patients with low, B with normal and C with high IGF-1 levels.

### IGF-1 determinations

IGF-1 was tested immunochemiluminometric by commercial assay (LabCorp, Burlington, NC), with normal range for all ages defined as 78–270 ng/mL.

### Main outcome measures

Because our center, based on patient age, low ovarian reserve, prior IVF cycles at other centers, and other adverse patient parameters, likely, serves the most adversely selected patient population among IVF centers in the USA (and possibly worldwide), the primary chosen endpoint for the study was cycle cancellations, likely the most sensitive endpoint among patients with high cycle cancellation rates. Secondary study end points were number of oocytes retrieved, embryos transferred, pregnancies, and live births. Because of low expected pregnancy rates, the study was, however, considered underpowered to consider them as primary endpoints. Primary and secondary endpoints were also investigated adjusted for patient age at time of presentation. The diagnosis of a clinical pregnancy mandated visualization of pregnancy on vaginal ultrasound examination.

### IVF cycle protocol

As already noted, our center serves a very homogenous, poor-prognosis patient population, characterized by advanced female age, large numbers of prior cycle failures, low functional ovarian reserve, and, therefore, ovarian resistance to stimulation. Patients, consequently, receive individualized ovarian stimulation protocols, which contain the following common denominators: (i) Every woman above age 40 and women below age 40 with LFOR for age and low peripheral androgen levels and/or elevated sex hormone binding globulin (SHBG) receives as previously reported, at least 6–8 weeks of pre-supplementation with dehydroepiandrosterone (DHEA) and CoQ10 prior to IVF cycle start [22]. DHEA supplementation is initiated only after baseline bloods, including IGF-1, are drawn. Cycles are initiated only once androgen levels and SHBG are in normal range. (ii) All cycles are initiated on days 2–3 of menses after ca. 10 days of luteal estrogen supplementation for priming purposes. (iii) Except in younger women with still adequate ovarian reserve, who, per Surrey et al. [23] receive a micro-dose agonist protocol, most patients receive ovarian stimulation without either agonist or antagonist since they receive HIER (highly individualized egg retrieval), with human chorionic gonadotropin (hCG) trigger of 10,000 IU, depending on female age and prior cycle history, at 12–16-mm lead follicle size [24, 25]. Because of the early egg retrieval, agonists/antagonists to prevent spontaneous ovulation are not required in such patients. (iv) All patients receive gonadotropin stimulation of 450–600 IU per day, usually at 3:1 ratio of FSH to human menopausal gonadotropin (hMG) products (manufacturers vary, depending on patient preference and/or insurance coverage). If patients have a history of very poor prior response to such stimulation, they in parallel also receive Clomiphene citrate 100 mg for 5 days, starting on day 2 of menses. (v) Considering the importance of every embryo in this patient population, the embryology laboratory performs, as also previously reported, rescue in vitro maturation of every immature oocyte [26]. (vi) Cryopreservation of embryos is as much as possible avoided and patients preferably undergo fresh transfers. (vi) Cycles utilizing autologous oocytes are always transferred at cleavage stage and transfers are performed under ultrasound control. (vii) Pregnancy test is obtained 12 days following embryo transfer.

### IRB approval

Since this study only involved data extraction from our center’s anonymized electronic medical research data base, it only required expedited IRB approvals. Every included patient provided written permission by consent to utilize their medical records for research purposes, as long as their anonymity was maintained, and the medical record remained confidential.

### Statistical analyses

Continuous variables were presented with mean ± standard deviation and compared between IGF-1 groups by an ANOVA test. Categorical variables were compared between IGF-1 groups with Fisher’s exact test. Age was compared to continuous IGF-1 levels by linear regression. Logistic regression and negative binomial regression models were used to adjusted for patients’ age. A *P*-value < 0.05 was considered statistically significant. Analyses were performed by the center’s medical statistician (S.K.D.) using SAS version 9.4 (SAS Institute, Cary, NC).

## Results

**Fig. 1:**
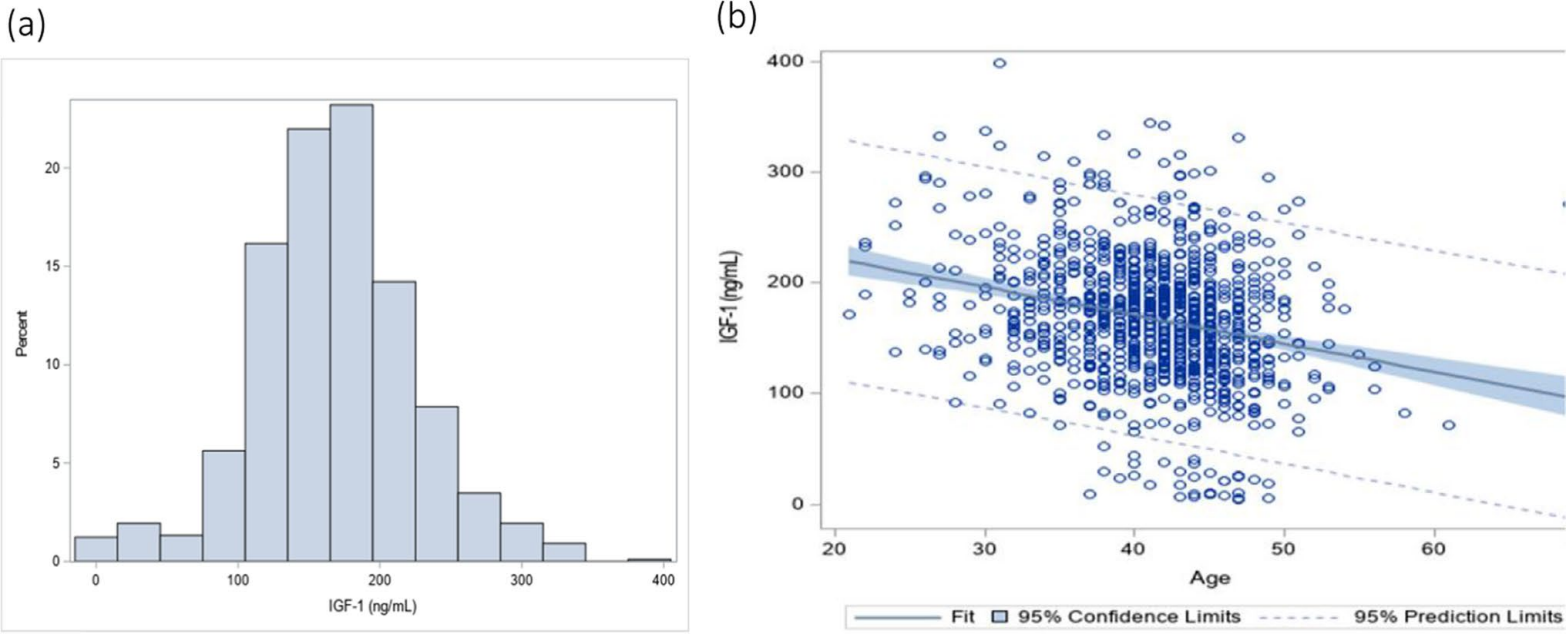
**a** Demonstrates the Gaussian distribution of IGF-levels in the study population. **b** Reflects the linear progression, demonstrating the age dependency of IGF-1 (*P* = 0.0007)

The distribution of IGF-1 levels in the whole study population was Gaussian (Fig. 1a). Patients in the lowest IGF-1 quartile (group A) were significantly older (43.0 ± 4.8 years) than those in mid-range (group B, 41.3 ± 4.9 years) and highest quartile group C (40.7 ± 5.6 years; *P* = 0.019). This is of importance because, as one would expect, IGF-1 levels were age dependent: A linear regression revealed that IGF-1 levels decreased with increasing age 2.2 ± 0.65 ng/mL per year (*P* = 0.0007; Fig. 1b).

Table 1 demonstrates further details: Though not statistically different, trends reflecting ovarian reserve parameters were the best in group B: FSH was 17.3 ± 17.8 vs. in group A, 24.8 ± 35.3 and in group C, 18.1 ± 20.6 mIU/mL; *P* = 0.085; AMH was 1.4 ± 3.3 vs. in group A, 0.7 ± 1.2 and in group C, 1.0 ± 1.6; *P* = 0.200).

**Table 1:** Patient differences between groups A, B, and C

	Group A	Group B	Group C	*P*-value	*P*-value adjusted for age
*N*	64	164	74		
Age (years)	43.0 ± 4.8	41.3 ± 4.9	40.7 ± 5.6	0.0191	
AMH (ng/mL)	0.7 ± 1.2	1.4 ± 3.3	1.0 ± 1.6	0.1995	
FSH (mIU mL)	24.8 ± 35.3	17.3 ± 17.8	18.1 ± 20.6	0.0845	
Cycles					
Cancelled cycles	16 (25.0%)	10 (13.5%)	9 (11.6%)	0.0421	0.0212
Oocytes retrieved	3.6 ± 5.4	5.2 ± 5.4	4.5 ± 5.0	0.1274	0.1870
Embryos transferred	1.1 ± 1.5	1.5 ± 1.4	1.4 ± 1.5	0.1668	0.1184
Pregnancies (%)	2 (3.1%)	9 (5.5%)	4 (5.4%)	0.8258	0.8518
Live births (%)	0 (0%)	7 (4.3%)	2 (2.7%)	0.2796	0.7765

Cycle cancellations were statistically the lowest in C (11.6%), the highest in A (25.0%), and in mid-range in B (13.5%; *P* = 0.042). Oocyte numbers, transferred embryos, pregnancy, and live birth rates did not differ significantly, though oocyte numbers trended the highest in group B (5.2 ± 5.4 years) vs. 3.6 ± 5.4 in group A and 4.5 ± 5.0 in group C. Adjusting statistical assessments for age, the difference in cancelled cycles became even more significant (*P* = 0.021), while all other outcome, likely because of too small patient numbers, remained non-significant.

## Discussion

It is important to initiate the discussion of here presented results by pointing out one more time the highly unfavorable selection of here presented patient population (Table 1). Not only were patients of advanced age, from a mean of 40.7 years in group C, 41.3 years in group B to a mean of 43.0 years in group A (*P* = 0.019), but they also demonstrate highly unfavorable functional ovarian reserve parameters, with FSH in this case demonstrating the best abnormal median in group B at 17.3 mIU/mL, group C with FSH 18.1mIU/mL holding the middle, and group A with 24.8 mIU/mL being the worst, though differences did not reach significance (*P* — 0.085). They, however, correlated with abnormally low AMH levels, with group B again demonstrating the best mean level of 1.4 ng/mL, followed by group C at 1.0 ng/mL and group A again demonstrating the by far poorest mean value at 0.7 ng/mL, though these differences were statistically also not significant.

Despite quite a large number of first IVF cycles (at our center) in this study (*n* = 302), because of the unfavorable prognosis of here investigated patient, pregnancy and live birth rates were as expected relatively low (Table 1). This can be assumed to be a reason why oocyte numbers retrieved, numbers of transferrable embryos and pregnancy, and live birth rates did not reach statistical significance between study groups. Cycle cancellation rates, clearly the most sensitive outcome parameter in poor prognosis patients, however, did demonstrated statistically significant differences between study groups based on IGF-1 level and these differences even strengthened with age adjustment. Further studies, involving even larger patient numbers as well as better prognosis patients, will, however, be helpful in reaching more definite answers as to why, even in most unfavorable IVF patients, cycle cancellations do statistically relate to IGF-1 levels.

Since cycle cancellation rates in this study clearly inversely correlated with IGF-1 levels, this study for the first time offers a potential selection tool for women in infertility treatments who may benefit from GH supplementation in association with IVF. All evidence points toward women in group B (normal IGF-1 levels) demonstrating best outcomes. This finding, alone, supports the study’s initial hypothesis that GH supplementation may improve IVF outcomes only in patients with low IGF-1 levels (group A). These findings potentially also explain the very conflicting results in the literature regarding GH utilization in association with IVF, as unselected utilization will, of course, dilute effectiveness of GH treatment: Just as aspirin will relive headache only in patients with headache and will be ineffective in a general population without a preponderance for headache, so will GH only be effective in women with low IGF-1 levels, through which GH exerts its physiological effects on ovaries.

Our results to a degree contradict studies from a single laboratory, claiming in two studies poorer IVF cycle outcomes with increasing IGF-1 levels [10, 27]. The same group in an earlier study, however, as we do here, reported highest cycle cancellations with lowest IGF-1 and lowest cancellations with highest IGF-1 [9]. Their most recent study involved so-called poor-responders but ages were clearly younger and FSH and AMH levels more favorable than in our patient population [27]. In addition, these authors defined high IGF-1 levels as anything over 72.0 ng/mL, while in our study, even the lowest 25th percentile was going as high as 132 ng/L. These two studies, therefore, are not comparable and an insightful accompanying editorial noted that drawing conclusions from this group’s recent study for several additional reasons was difficult [28]. For these reasons and because of physiological logic, here observed statistical correlation between low IGF-1 and increased IVF cycle cancellation risk, therefore, is credible.

At absolute minimum, GH supplementation, thus, appears indicated in women with low peripheral IGF-1 levels, in this study defined as < 132 ng/mL (lower 25th percentile). As noted earlier in the “Introduction” section of this manuscript, positive effects of GH supplementation should not surprise in absence of GH and especially of adequate IGF-1 levels [13, 18–20]. Though such supplementation has remained controversial [5, 6], our improving understanding of GH/IGF-1 effects on granulosa cells and the resulting synergism with FSH effects on follicle growth support such supplementation but only if it occurs in women with low IGF-1 levels. We, therefore, propose that future studies of GH supplementation in IVF cycles should be preceded by IGF-1 evaluations and only women with abnormally low levels should be considered for such supplementations.

Here presented findings are, however, also interesting for their apparent contradictions: On the one hand, there appears strong evidence for a beneficial effect of IGF-1 on IVF cycle completion; yet, while the positive effect on cycle completion appears linear with increasing IGF-values, functional ovarian reserve, as represented by FSH and AMH levels, on the other hand, appears best at mid-levels of IGF-1 (group B). Cycle cancellations as well as FOR are clearly the worst in group A, also the oldest patients in this study and, therefore, are not a surprise. Reaffirming the likelihood of a causal association with IGF-1, age, however, does not appear to explain cycle cancellations since significance was maintained (and actually improved) after age adjustments (*P* = 0.021).

Cycle cancellations automatically denote IVF cycle failure. Though a statistical association does not establish causation, here demonstrated statistical association between IGF-1 levels and IVF outcomes strongly supports a causal relationship since this association even strengthened after age adjustments. How, specifically, IGF-1 lowers cycle cancellation risks, remains to be established. Cycle completion mandates at least one oocyte and one transferrable embryo. One, therefore, may conclude from here presented findings that better IGF-1 levels support the likelihood that at least one embryo becomes available for transfer. IGF-1, may achieve this by, as previously noted, enhancing recruitment [29] and acting synergistically with androgens and FSH in follicle maturation during small growing follicle stages [21]. Improvements in egg and embryo numbers after GH supplementation have, indeed, also been reported in studies that have failed to demonstrate improvements in pregnancy and live birth rates [5, 6] and, therefore, based on existing literature appear as of this point factual.

Whether there are other ways by which the GH-IGF-1 axis may beneficially influence IVF outcomes remains as of this point unsettled. An aged mouse model, recently reported by Chinese investigators, offers complementary information to here presented data: In that study, the authors confirmed that GH increased the number of antral follicles and of retrieved oocytes most at a medium dosage, second-best at high dosage and least at low dosage. This effect was achieved in those animals without obvious changes in AMH levels. Because improvements also correlated with increasing ATP levels, frequency of homogenous mitochondrial distribution, and improved mitochondrial membrane potential (though not with mtDNA copy numbers), the authors suggested that GH improved mitochondrial function in oocytes [30]. GH, in addition, also appears effective in improving in vitro maturation of human oocytes [31, 32]. Finally, recent studies also strongly hint at effects of GH on endometrial receptivity [32] which, thus, potentially appears to offer an independent contribution to improved IVF outcomes from ovarian HGH/IGF-1-effects.

After female age, egg and embryo numbers in a given IVF cycle represent the second most-important predictor of pregnancy and live birth chances in IVF [33]. They in that same study also related in a rather peculiar way to AMH levels that may also have some relevance to here reported results: As pregnancy and live birth rates increased with larger egg and embryo yields, they did so also in parallel to increasing AMH levels. That increase, however, persisted only up to a certain AMH level, at which point, with further increasing AMH, not only did pregnancy rates start declining but miscarriage rates skyrocketed. Beyond certain AMH threshold-levels, its initially positive effects on IVF outcomes, thus, turned radically negative. IGF-1 may demonstrate a similar effect-reversal with increasing concentrations in the peripheral circulation, as Irani et al. in frozen-thawed IVF cycles recently reported higher miscarriage rates associated with higher peripheral IGF-1 levels [34]. This observation further supports above noted suspicion that this group of investigators dealt with a very different patient population with quite different IGF-1 cut offs in comparison to this study,.

Here presented IGF-1 data, suggesting best FOR at mid-range for IGF-1 (in our study at roughly 132–202 ng/mL), are supported by above noted mouse study [30], suggesting similar IGF-1 dynamics, with a “best” level at mid-range. Endocrinology is defined by “best” endocrine ranges for practically all hormones. Another good example in control of ovarian function is androgen levels, with too low and too high, producing subpar IVF outcomes [21].

## Limitations, summary, and conclusions

The highly unfavorable patient populations our center serves obviously limits the applicability of here reached conclusions (Table 1). Considering the advanced age and low functional ovarian reserve of all three here reported patient groups, IVF outcomes were characterized by relatively small oocyte yield, embryo numbers, and few pregnancies. Consequently, it is not surprising that, despite a reasonably large patient population, no significant differences were observed in secondary IVF cycle outcome parameters. Though difficult to assess considering the various outcome parameters, our statistician concluded that study groups would at least have to double in size to also demonstrate differences in other clinical IVF cycle outcome parameters than cycle cancellations. Though advanced ages of the study population must be carefully considered before generalizing here observed findings to younger age groups, that age adjustment actually improved the significance of here reported finding, in a way validates them.

This study clearly supports further exploration of GH supplementation especially in women with low IGF-1 levels, usually mostly older patients. While ovaries in younger women may reveal different hormonal dynamics, we, therefore, would not be surprised if younger women with low IGF-1 levels would also be positively affected by supplementation with GH.

Two additional issues deserve mention: As IGF-effects on ovaries are most profound at small growing follicle stages, follicles exposed to adequate IGF-1 levels still require at least 6–8 weeks to reach gonadotropin-dependence that renders them available to gonadotropin stimulation in IVF cycles. GH supplementation must, therefore, be started at least 6–8 weeks before IVF cycle start. A large majority of studies in the medical literature supplemented patients with GH, however, only during stimulation or, at best, starting about 2 weeks before stimulation start. Such supplementation, like androgen supplementation which supports follicle growth with identical timing [21], will not result in desired effects on granulosa cells of growing follicles (and, therefore, oocytes), though they may, at right concentrations, exert beneficial endometrial effects [35, 36]. Second, the literature also varies greatly in daily dosages of GH that were administered. Here, too, a consensus must be reached if study outcomes are to be compared.

As a final message, this manuscript also suggests that determination of IGF-values, generally not considered a routine test in infertility practice, may be indicated in women with low functional ovarian reserve.

## Data Availability

Data and materials are upon reasonable request available from The CHR’s data depository by contacting Ms. Jolanta Tapper, COO at jtapper@thechr.com/.
